# Multidimensional Assessment of Strengths and Their Association With Mental Health in Psychotherapy Patients at the Beginning of Treatment

**DOI:** 10.32872/cpe.8041

**Published:** 2023-06-29

**Authors:** Jan Schürmann-Vengels, Stefan Troche, Philipp Pascal Victor, Tobias Teismann, Ulrike Willutzki

**Affiliations:** 1 Department of Psychology and Psychotherapy, Witten/Herdecke University, Witten, Germany; 2 Department of Psychology, University of Bern, Bern, Switzerland; 3 Mental Health Research and Treatment Center, Ruhr-Universität Bochum, Bochum, Germany; Philipps-University of Marburg, Marburg, Germany

**Keywords:** strengths, resources, resilience, mental health, dual-factor model, structural equation model

## Abstract

**Background:**

Modern concepts assume that mental health is not just the absence of mental illness but is also characterized by positive well-being. Recent findings indicated a less pronounced distinction of positive and negative mental health dimensions in clinical samples. Self-perceived strengths were associated with markers of mental health in healthy individuals. However, analyses of strengths and their association with different mental health variables in clinical populations are scarce.

**Method:**

A cross-sectional design was conducted at a German outpatient training and research center. 274 patients before treatment (female: 66.4%, mean age = 42.53, SD = 13.34, range = 18-79) filled out the Witten Strengths and Resource Form (WIRF), a multidimensional self-report of strengths, as well as other instruments assessing positive and negative mental health variables. Data was analyzed with structural equation modeling and latent regression analyses.

**Results:**

Confirmatory factor analysis of the WIRF showed good model fit for the assumed three-subscale solution. Regarding mental health, a one-factor model with positive and negative variables as opposite poles showed acceptable fit. A correlated dual-factor model was not appropriate for the data. All WIRF subscales significantly predicted unique parts of variance of the latent mental illness factor (p = .035 – p < .001).

**Conclusion:**

The context-specific assessment of patients’ strengths was confirmed and led to an information gain in the prediction of mental health. Results suggest that positive and negative facets of mental health are highly entwined in people with pronounced symptoms. The scientific and practical implications of these findings are discussed.

## Highlights

- The Witten Strengths and Resource Form (WIRF) captures strengths in three situational contexts.
- A confirmatory factor analysis supported the context-structure of the WIRF in a clinical sample.
- Positive and negative mental health variables were highly correlated in patients before treatment.
- WIRF subscales provided incremental information in the prediction of patients’ mental health.

Traditionally mental health has been understood as the absence of psychopathology. This view suggests that people are either mentally ill or mentally healthy at a given point in time. In contrast, modern dual-factor models emphasize a two-dimensional structure of mental health (Keyes, 2002; WHO, 2005). According to such models, a dimension of negative mental health (NMH) is defined by the absence or presence of mental illness and burden, whereas a positive mental health (PMH) dimension is characterized by high or low emotional, psychological, and social well-being. In contrast to the unidimensional view of mental health, two-factor models assume that these two dimensions are negatively related but still distinct from each other (Iasiello et al., 2020; Keyes, 2005). On the one hand, this means that individuals with mental disorders can still have moderate to high levels of well-being. On the other hand, a person with low well-being may not necessarily develop psychopathology. These assumptions were examined using various statistical approaches in healthy samples (Iasiello et al., 2020). In most studies, both dimensions were assessed with specific instruments and then examined with confirmatory factor analysis or structural equation models (SEM). These procedures are used when created theoretical models are to be tested with empirical data (Schreiber et al., 2006). Latent factors, such as mental health, that cannot be measured directly are extracted from the observed data. This allows a way to determine whether the study participants' data are more consistent with a one-dimensional or a two-factor understanding of mental health. Findings with healthy samples consistently showed that a model with two correlated factors (NMH and PMH) best reflects mental health (Kim et al., 2014; Magalhães & Calheiros, 2017). This result means that psychopathology is only on average and not necessarily associated with lower well-being. If NMH and PMH are at least partially distinct factors, it may be useful to examine specific correlates and predictors of these two dimensions (Schotanus-Dijkstra et al., 2017).

Findings from clinical samples showed mixed results for the dual-factor hypothesis. Most studies also found evidence for a correlated two-dimensional model of mental health (Alterman et al., 2010; de Vos et al., 2018; Díaz et al., 2018; Franken et al., 2018; Teismann et al., 2018; Tomba et al., 2014). On the other hand, van Erp Taalman Kip and Hutschemaekers (2018) showed that only a one-factor model of mental health fitted the data in an outpatient sample (*n* = 1069). The authors stated that psychopathology and well-being were more entwined in people with pronounced symptoms than in healthy subjects. This would imply that high psychopathology is almost always connected with low well-being (van Erp Taalman Kip & Hutschemaekers, 2018). One possible reason for this may be that people with mental disorders experience high levels of negative affect, meaning they often feel bad in everyday life (Stanton & Watson, 2014). This, in turn, could make it more difficult to feel good about potentially pleasant experiences or situations (Carl et al., 2013). Such limited positive reactivity might prevent individuals with marked psychopathology from also feeling well (at least temporarily). Statistically, such a global perception by patients of either feeling bad or good is expressed in a high negative correlation between psychopathology and well-being. Various studies, including the ones that found evidence for a dual-factor structure of mental health, found large correlations of NMH and PMH measures in clinical samples, *r* = -.67 – -.72 (Bos et al., 2016; Franken et al., 2018; Lukat et al., 2016; van Erp Taalman Kip & Hutschemaekers 2018). These correlations are significantly higher than in healthy individuals, suggesting that patients may have less access to or less acknowledge positive experiences and situations at the beginning of psychotherapy because these are overshadowed by high symptom burden (Iasiello et al., 2020). In turn, this makes it difficult for clinicians to utilize the positive experience of patients in psychotherapy.

Psychological strengths (also named resources; Munder et al., 2019) are discussed as promotive factors of mental health for both healthy and clinical samples (Grawe & Grawe-Gerber, 1999; Taylor & Broffman, 2011). Strengths are defined as already existing intra- and interpersonal potentials and abilities of a person (Grawe, 1997; Willutzki, 2008). Several authors argued that an aspect is defined as a strength by the following criteria: (1) subjective positive evaluation, and/or (2) functionality to reach personal goals (Grawe 1997; Willutzki, 2008). The literature often distinguishes personal and social strengths (Taylor & Broffman, 2011). Examples of personal strengths are the optimistic handling of difficulties and the implementation of individually positive activities, while social strengths are characteristics that help to form good relationships or perceive contacts. Current concepts of strengths point to the importance of situational context in judging whether an aspect is positive and/or helpful (Flückiger, 2009; Taylor & Broffman, 2011; Willutzki, 2008). For example, a supporting family member or friend can be a highly important resource to cope with everyday problems. However, a supporting person may also be part of the avoidance system of an anxiety disorder, making approach coping more difficult in this specific situation. Research findings further indicated that aspects, rated as strengths by the person him-/herself (self-perceived strengths), are stronger related to good mental health outcomes compared to observer rated factors (Melrose et al., 2015; Prati & Pietrantoni, 2010). Various studies showed that self-perceived strengths were strongly associated with higher PMH and predicted participants’ long-term well-being in healthy samples (Gloria & Steinhardt, 2016; Mc Elroy & Hevey, 2014; Niemeyer et al., 2019; Siedlecki et al., 2014).

Strengths and their relationship to mental health are less researched in clinical populations, although the activation of strengths is a widely supported mechanism in psychotherapy (Munder et al., 2019). It is assumed that people with mental disorders often do not perceive possible strengths in themselves as strengths, although these are recognized as such by outsiders (For example, the therapist values the patient's creativity as helpful, while the patient perceives it as trivial for coping with the problem). High levels of psychopathology appear to be associated with negativity biases, which may be one reason why patients have less access to their own strengths that are present despite their distress (Stanton & Watson, 2014; Trompetter et al., 2017). With respect to this, two studies showed that both psychiatric inpatients and psychotherapy outpatients report significantly lower levels of self-perceived strengths compared to healthy individuals with large effect sizes for this difference (Goldbach et al., 2020; Victor et al., 2019). Most available instruments assess strengths over all situations a person experiences (trans-situational). Such global measures can be problematic in clinical samples because they only reflect that patients have a strong focus on their problems and, in turn, a low perception of their strengths (Iasiello et al., 2022; Joseph & Wood, 2010). Thus, such instruments do not provide additional information compared to problem measurements in the clinical context.

Therefore, Victor et al. (2019) developed the Witten Strengths and Resource Form (WIRF), an assessment tool designed to capture strengths in three situational contexts: (1) strengths in everyday life (EvdayS), (2) strengths used to successfully cope with previous crises (CrisesS), and (3) strengths in connection with current problems (ProbS). The multidimensional structure was transferred from an existing diagnostic interview and obtained for the questionnaire by means of an exploratory factor analysis using data from a sample of 144 psychotherapy patients (Victor et al., 2019; Willutzki et al., 2005). To determine construct validity, the subscales were correlated with relevant instruments: All subscales showed significant positive correlations with an established strengths instrument (Tagay et al., 2014; Victor et al., 2019). The instrument is designed to capture how patients rate their strengths in dealing with different situations. A person may indeed have different thoughts about how pronounced and helpful one's strengths are in different circumstances, so that diverse aspects of patients' perceptions could be represented by the subscales of the WIRF. For example, people who are currently under a lot of stress, but at the same time know what strengths have helped them in the past, may feel more able to manage the difficulty. The inclusion of different subscales of the WIRF would amount to incremental prediction of, for example, mental health, because the subscales contain different information of patients’ experience. However, whether the subscales of the WIRF capture different aspects of strengths perception is still unclear and needs to be confirmed confirmatory in a larger sample.

## Objectives

To the best of our knowledge, no study has yet analyzed the association of strengths with different mental health variables in the clinical context. The first aim of this study was to confirm the three-subscale structure of the WIRF in a sample of psychotherapy outpatients. Furthermore, to extend research on the dual-factor model, the second aim was to analyze the latent factor structure of mental health in psychotherapy outpatients with different positive and negative measures. The third aim of this study was to explore whether the strengths subscales of the WIRF may predict unique parts of patients’ mental health/mental illness.

*H1:* It is expected that the structure of the WIRF with (1) strengths in everyday life (EvdayS), (2) strengths used to successfully cope with previous crises (CrisesS), and (3) strengths in connection with current problems (ProbS) as separate subscales will show a good model fit in a clinical sample.

*H2*: It is expected that a dual-factor model of mental health – with PMH and NMH as correlated, but distinct factors – will be a more appropriate description of mental health related data in a clinical sample compared to a one-factor model with PMH and NMH as opposite poles of the same dimension. To address this hypothesis, two latent factor models will be created based on actual measurements and tested against each other in terms of model fit.

*H3*: It is further hypothesized that all WIRF subscales will significantly predict unique variance in the latent factors of mental health/mental illness. For the EvdayS scale, small to moderate positive correlations are expected only with measures of PMH. For the CrisesS scale, small to moderate correlations are expected with measures of PMH (positively directed) and NMH (negatively directed). ProbS is expected to correlate strongly positive with PMH measures and strongly negative with NMH measures.

## Method

### Design and Sample Description

Participants were recruited between 2016 and 2019 at the Center of Mental Health and Psychotherapy (CMHP), an outpatient training and research center for cognitive behavioral therapy (CBT) at Witten/Herdecke University, Germany. A cross-sectional design was applied where patients filled out all instruments at one point in time before the first psychotherapy session. General inclusion criteria were as follows: (1) at least one mental disorder according to DSM-IV criteria, (2) at least 16 years of age, (3) sufficient German language skills. Patients that fulfilled inclusion criteria were informed about the study procedures and signed the informed consent. After study inclusion, patients’ diagnoses were determined with the Structured Clinical Interview for DSM-IV (SCID; Wittchen et al., 1997) within the first treatment sessions. Diagnostic interviews were performed by licensed CBT therapists or trainee therapists in advanced CBT training. All therapists were trained in the use of diagnostic interviews in prior workshops as a part of their training schedules.

The total sample consisted of 274 adult psychotherapy outpatients (female: 66.4%, *M_age_* = 42.53, *SD* = 13.34, range = 18-79). Most common primary diagnoses were affective disorders (33.58%), anxiety disorders (17.88%), and adjustment disorders (12.04%). 33 patients (12.04%) had at least two disorders. On average, patients had 1.14 diagnoses (*SD* = 0.40, range: 1-3). More than half the patients (52.55%) had prior psychological treatment. Table 1 shows demographic data of the clinical sample.

**Table 1:** Description of the Clinical Sample

Characteristic		
	*M*	*SD*
**Age**	42.53	13.34
	*n*	%
Gender		
Female	182	66.42
Male	86	31.39
Missing	6	2.19
Relationship statusᵃ		
Single	80	29.20
In a relationship	146	53.28
Level of educationᵃ		
No graduation	4	1.46
Secondary education	56	20.44
A levels	46	16.79
Academic degree	36	13.14
Completed apprenticeship	122	44.53
Employmentᵃ		
Employed	164	60.00
Self-employed	7	2.55
Unemployed	49	17.88
Training/Studies	3	1.09
Retired	29	10.58

^a^optional answer.

### Instruments

#### Self-Perceived Strengths

Patients’ strengths were assessed with the WIRF (Victor et al., 2019). The instrument conceptualized strengths as individually usable abilities that help to cope with specific situations (Munder et al., 2019; Taylor & Broffman, 2011). The WIRF is a multidimensional self-report with 36 items (Likert scale from 0 “completely disagree” to 5 “completely agree”), assessing a person’s strengths with three subscales: strengths in everyday life (EvdayS), strengths in previous successful crises management (CrisesS), and strengths in connection with current problems (ProbS). Participants are presented with various strengths and asked to what extent they were able to use them in the specific context. In each subscale, the same 12 items are presented in a different order to compare a person's perception of strengths across contexts. Each subscale starts with a short introduction referring to the context (e.g., for CrisesS: In the next step we would like to ask you to think back to rather difficult times of your life. Everybody goes through such times. Please now think of a situation that was difficult for you to handle, but which you nevertheless tackled successfully, i.e., a situation about which you would say today: “I handled that pretty well”, or “I’m quite happy with myself about how I did that”. The following statements suggest some possible actions people can take in difficult situations).

A mean score was calculated for each subscale, which represents a patient’s global perception of whether he/she experiences his or her existing strengths as sufficient and helpful in the respective context. Items can be further grouped into three themes: action regulation (planning and performing activities), relaxation (taking time to relax and enjoy life), and social strengths (helpful interaction patterns).

The WIRF was developed based on a multidimensional concept from an existing diagnostic interview (Willutzki et al., 2005). A survey of psychotherapy experts, identifying relevant strengths, was conducted to create an item pool. After this, a preliminary strengths questionnaire was developed and tested in a sample of psychotherapy outpatients different from the one in this study (*n* = 144), yielding to the WIRF. Item indices as well as psychometric properties were analyzed in both a clinical sample and healthy controls (Victor et al., 2019). All subscales showed good internal consistency (α = .84 – .88). Moreover, the subscales showed hypothesis-consistent correlations with other strengths and social support assessments, indicating convergent validity (Victor et al., 2019).

#### PMH Constructs

##### The WHO-5 Well-Being Index

The WHO-5 (Bech et al., 2003; WHO, 1998) is an internationally used five item self-report to assess the general subjective well-being of a person in the last two weeks (Likert scale from 0 “At no time” to 5 “All the time”). Subjective well-being is characterized by the frequency of positive feelings and one’s satisfaction with life (Topp et al., 2015). A mean score of the five items was used to represent a person’s general well-being in this study. The German version showed excellent internal consistency, α = .92 (Brähler et al., 2007). Moreover, a systematic review indicated good construct and predictive validity of the instrument in healthy and clinical samples (Topp et al., 2015). Internal consistency in our sample was α = .88.

##### The Sense of Coherence Scale – Short Form

The SOC-L9 (Schumacher et al., 2000) assesses a person’s sense of coherence as conceptualized in the salutogenic model of health (Antonovsky, 1987). Sense of coherence is operationalized by three components (comprehensibility, manageability, meaningfulness) and describes the global orientation of an individual that he/she has the resources to cope with stress and life in general (Antonovsky, 1987). The instrument contains nine items (Likert scale from 1 “Very often” to 7 “Rarely/Never”), from which a mean score is formed that reflects the global sense of coherence. The German version showed good internal consistency, α = .87 (Singer & Brähler, 2007). Another study showed evidence for construct validity of the SOC-L9 with significant correlations with established PMH scales, *r* = .60 – .64 (Lin et al., 2020). Internal consistency in our sample was α = .85.

#### NMH Constructs

##### The Brief Symptom Inventory – Short Version

The BSI-18 (Spitzer et al., 2011) is a self-report measure to assess psychopathology in the last week. It contains 18 items (Likert scale from 0 “Not at all” to 4 “Nearly every day”), measuring symptoms of somatization, anxiety, and depression. The global severity index (GSI) of the instrument was used to represent a person’s level of general psychopathology in this study. Internal consistency of the GSI was good to excellent in several clinical samples, α = .88 – .93 (Franke et al., 2017; Spitzer et al., 2011). Internal consistency in our sample was α = .89.

##### The Perceived Stress Questionnaire

The PSQ-20 (Fliege et al., 2001) is an internationally used self-report measure to assess stress experience in the last four weeks. Stress is operationalized by four components (tension, worries, overload, lack of joy) and represents the global level of current burden. The instrument contains 20 items (Likert scale from 1 “Almost never” to 5 “Usually”), that were averaged to a mean score in this study. The German version showed good internal consistency, α = .80 – .86 (Fliege et al., 2001). Moreover, evidence of construct validity was indicated with negative associations with quality of life and social support measures (Fliege et al., 2001). Internal consistency in our sample was α = .92.

##### The Incongruence Questionnaire – Short Version

The K-INK (Grosse Holtforth & Grawe, 2003) is a self-report assessing psychological incongruence resulting from an insufficient realization of motivational goals. A high level of incongruence occurs when a person’s real-world experiences do not match with their desired goal states. The authors stated that incongruence is closely related to the experience of psychopathological symptoms (Grosse Holtforth & Grawe, 2003). It consists of 23 items (Likert scale from 1 “Far too little” to 5 “Perfectly good”) measuring incongruence in the context of both approximation and avoidance. A mean score was formed from the 23 items representing global incongruence. The German version showed good to excellent internal consistency in clinical samples, α = .87 – .91 (Grosse Holtforth & Grawe, 2003). Internal consistency in our sample was α = .89.

### Statistical Analyses

All analyses were conducted using R, version 3.6.3, packages: lavaan (Rosseel, 2012). Descriptive statistics of sample characteristics and analyzed variables were determined. Normality of analyzed variables was tested with separate Shapiro-Wilk’s tests. Bivariate correlations between analyzed variables were determined and tested with a significance level of α = .05.

In order to examine the main hypotheses, SEM using maximum likelihood estimation with robust standard errors (Huber-White) and scaled test-statistics were conducted (MLR; Rosseel, 2012). This procedure allows constructs that are not directly observable to be derived from the data (latent factors) and placed in relation to one another (Schreiber et al., 2006). Goodness of fit for all models was evaluated with a combination of well-established fit indices: comparative fit index (CFI), root mean square of approximation (RMSEA), standardized root mean square residual (SRMR). Hu and Bentler (1999) recommended the following criteria: CFI ≥ .95, RMSEA ≤ .06, SRMR ≤ .08 (good fit); CFI ≥ .90, RMSEA ≤ .08 (acceptable fit). Moreover, chi-square statistics for each SEM were determined. Several studies found that results of chi-square tests in SEM were highly related to sample size, therefore, it was not used for an interpretation of model fit in this study (Hu & Bentler, 1999; Peugh & Feldon, 2020).

**Figure 1:**
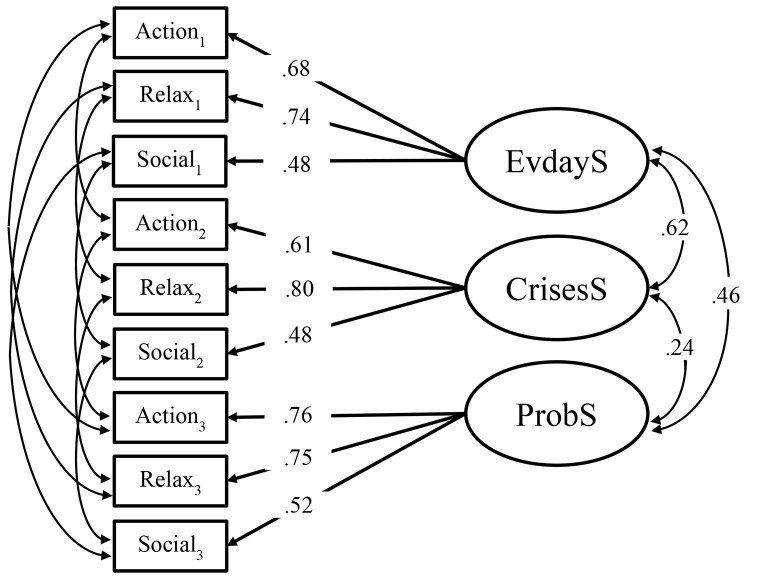
Structural Equation Model of the Three-Subscale Solution of the WIRF *Note.* EvdayS = Witten Strengths and Resource Form, strengths in everyday life; CrisesS = Witten Strengths and Resource Form, strengths used in prior crises; ProbS = Witten Strengths and Resource Form, in connection with current problems; Action/Relax/Social = Items of WIRF parceled to action regulation, relaxation, and social support.

To examine the first hypothesis, whether the subscales of the strengths instrument capture different facets, a SEM with the latent variables WIRF-EvdayS, WIRF-CrisesS, and WIRF-ProbS was arranged. Latent variables are usually defined with the single items of the respective measure. However, based on assumptions from prior studies, it was assumed that such a model would have included too many parameters and would have led to estimation problems with respect to the sample size (Little et al., 2002). Therefore, item parceling was used to reduce the number of parameters in this SEM. Parceling describes that a subset of items is bundled to packages. In this case, the single items were averaged to scores of the three strengths themes found by Victor et al. (2019): action regulation (5 items) relaxation (4 items) social strengths (3 items). Latent variables were defined with the item bundles in each context (see Figure 1). All latent variables were allowed to covary. Furthermore, residual covariances were allowed between corresponding manifest variables in the three subscales (e.g., relaxation in WIRF-EvdayS and WIRF-CrisesS).

To examine the second hypothesis, two measurement models for mental health were compared. The first model assumed a dual-factor structure with WHO-5 and SOC-L9 being indicators of a latent variable representing PMH and BSI-18, PSQ-20 and K-INK being indicators of a latent variable representing NMH. Latent variables were allowed to covary. The second model assumed a one-factor structure with all manifest variables loading on one latent variable. Models were compared with Akaike Information Criterion (AIC) to determine which model better fit the data. The AIC is used to compare nested models, with lower values indicating a better model fit (Boedeker, 2017).

To examine the third hypothesis, a SEM combining the better fitting model of mental health from Hypothesis 2 with the WIRF model from Hypothesis 1 was arranged. Stepwise regression analyses with the latent variables WIRF-EvdayS, WIRF-CrisesS, and WIRF-ProbS as predictors of the latent mental health/illness factor were conducted and tested with a significance level of α = .05.

## Results

### Preliminary Analyses

Total missing data was 4.93%. All analyzed variables but WIRF-ProbS showed deviations from the normal distribution, *p* = .028 – *p* < .001. Therefore, non-parametric correlations (Spearman) were determined for these relationships: WIRF subscales as manifest variables were significantly correlated with moderate to large coefficients, *r* = .35 - .60, *ps* < .001. All PMH and NMH variables were strongly correlated to each other. WIRF-EvdayS and WIRF-CrisesS showed modest correlation coefficients in their association with PMH and NMH variables. WIRF-ProbS was moderately to strongly correlated to PMH and NMH measures. Table 2 shows descriptive statistics and correlations of analyzed variables.

**Table 2:** Descriptive Statistics and Correlations of Analyzed Variables

Measure	1	2	3	4	5	6	7	8
1. WIRF-EvdayS	–							
2. WIRF-CrisesS	.60***	–						
3. WIRF-ProbS	.43***	.35***	–					
4. WHO-5	.19**	.18**	.55***	–				
5. SOC-L9	.16**	.24***	.42***	.50***	–			
6. BSI-18	-.12	-.14*	-.44***	-.56***	-.67***	–		
7. PSQ-20	-.11	-.13*	-.44***	-.58***	.67***	.61***	–	
8. K-INK	-.19**	-.17**	-.50***	-.53***	.75***	.59***	.68***	–
*M*	3.39^a^	3.00^a^	2.87^a^	1.62^b^	3.80^c^	1.13^d^	2.89^e^	3.05^f^
*SD*	0.82	0.91	0.94	1.00	1.13	0.72	0.56	0.66

*Note.* Spearman ρ coefficients are displayed; WIRF-EvdayS = Witten Strengths and Resource Form, strengths in everyday life; WIRF-CrisesS = Witten Strengths and Resource Form, strengths used in prior crises; WIRF-ProbS = Witten Strengths and Resource Form, strengths in connection with current problems; WHO-5 = WHO-5 Well-being Index; SOC-L9 = Sense of Coherence scale – Short form; BSI-18 = Brief Symptom Inventory – Short version; PSQ-20 = Perceived Stress Questionnaire; K-INK = Incongruence questionnaire – Short version.

^a^*n* = 274. ^b^*n* = 257. ^c^*n* = 258. ^d^*n* = 245. ^e^*n* = 243. ^f^*n* = 259.

*p < .05. **p < .01. ***p < .001.

### Measurement Models

The first step was to review the context structure of the WIRF. Although the chi-square test statistic was statistically significant, the other fit indices suggested that the three-subscale solution for the WIRF could be confirmed by means of confirmatory factor analysis, χ^2^_MLR_(15) = 28.43, *p* = .019, CFI = .98, RMSEA = .06, SRMR = .06. Although all WIRF subscales consist of the same items, three delineable factors could be filtered from the data. Thus, it seems warranted to assess strengths in the different contexts separately, since the subscales overlap only partially.

In a next step, the dual- and the one-factor model of mental health were computed and compared against each other. The model fit for the dual-factor model was good regarding CFI (.99) and SRMR (.02). However, χ^2^_MLR_-test statistic was significant, χ^2^(4) = 12.29, *p* = .015, and the RMSEA of .09 was too large. The AIC was 2275.38. Moreover, the covariance matrix of the latent variables in the dual-factor model was not positive definite due to a high estimated correlation between NMH and PMH suggesting virtual identity of the two latent variables.

**Figure 2:**
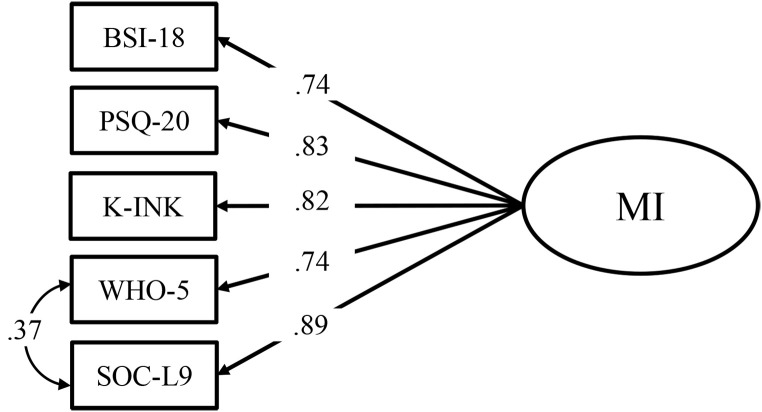
Structural Equation Model of the One-Factor Model of Mental Illness *Note.* MI = Latent mental illness factor; BSI-18 = Brief Symptom Inventory – Short version; PSQ-20 = Perceived Stress Questionnaire; K-INK = Incongruence questionnaire – Short version; WHO-5 = WHO-5 Well-being Index; SOC-L9 = Sense of Coherence scale – Short form.

The fit of the one-factor model, however, was worse compared to the dual-factor model, χ^2^_MLR_(5) = 25.54, *p* < .001, CFI = .97, RMSEA = .13, SRMR = .03, AIC = 2287.22. In sum, the dual-factor model led to estimation problems, but the one-factor model did not describe the data adequately. Therefore, we sought to improve the data description of the one-factor model, which could be achieved by allowing a residual correlation between the two indicators of PMH (i.e., WHO-5 and SOC). This led to a trending acceptable data fit of the one-factor model, χ^2^_MLR_(4) = 12.29, *p* = .015, CFI = .99, RMSEA = .09, SRMR = .02, AIC = 2275.38. Thus, confirmatory factor analysis revealed that a dual-factor structure for mental health with a differentiation between positive and negative aspects was not appropriate in our sample. The closest fit was a bipolar model (one factor) in which high mental illness was almost always associated with low mental health. The further analyses were conducted based on the adjusted one-factor model. The latent factor of this model will be named mental illness in the following, because NMH constructs loaded positively, while PMH constructs loaded negatively on that factor (see Figure 2).

### Latent Regression Analyses

**Figure 3:**
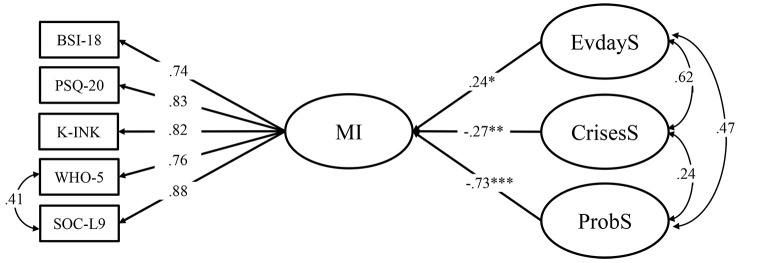
Core of the Structural Equation Model for the Regression of the WIRF Subscales on Mental Illness *Note.* EvdayS = Witten Strengths and Resource Form, strengths in everyday life; CrisesS = Witten Strengths and Resource Form, strengths used in prior crises; ProbS = Witten Strengths and Resource Form, in connection with current problems; MI = Latent mental illness factor; BSI-18 = Brief Symptom Inventory – Short version; PSQ-20 = Perceived Stress Questionnaire; K-INK = Incongruence questionnaire – Short version; WHO-5 = WHO-5 Well-being Index; SOC-L9 = Sense of Coherence scale – Short form.

After having established measurement models of strengths and mental health, we investigated the relationship between the WIRF subscales and general mental illness by means of a latent regression analysis (see Figure 3).

When mental illness was regressed on the three subscales of the WIRF separately, all three regression coefficients were statistically significant with β = -0.36, *p* = .007, for WIRF-EvdayS, β = -0.29, *p* = .007, for WIRF-CrisesS, and β = -0.67, *p* < .001, for WIRF-ProbS.

The model resulting from the multiple regression of mental illness on all three WIRF subscales fitted the data well, χ^2^_MLR_(61) = 126.12, *p* < .001, CFI = .96, RMSEA = .06, SRMR = .05. WIRF-CrisesS and WIRF-ProbS were almost unchanged when compared to the single regression analyses. More self-perceived strengths in these contexts were associated with less mental illness. The two scales are incrementally significant and predict independent proportions of mental illness. However, the link between mental health and WIRF-EvdayS changed its sign from negative to positive. This may be interpreted as a negative suppression effect resulting from the inclusion of other predictors (Beckstead, 2012). In a post-hoc analysis, it was found that the inclusion of WIRF-ProbS affected this suppression effect on WIRF-EvdayS, suggesting that these two subscales share a high common intersection with the criterion (mental illness). WIRF-EvdayS can, therefore, not be considered an independent predictor. Table 3 shows results of the latent regression analysis.

**Table 3:** Results of the Latent Regression Analysis With All WIRF Subscales Included as Predictors

Variables	*b*	*SE*	*z*	*p*	Std.lv
Criterion: Mental illness^a^					
WIRF-EvdayS	0.20	0.10	2.11	.035	0.24
WIRF-CrisesS	-0.21	0.08	-2.62	.009	-0.27
WIRF-ProbS	-0.44	0.05	-8.69	< .001	-0.73

*Note.* WIRF-EvdayS = Witten Strengths and Resource Form, strengths in everyday life; WIRF-CrisesS = Witten Strengths and Resource Form, strengths used in prior crises; WIRF-ProbS = Witten Strengths and Resource Form, in connection with current problems; b = estimate of predictor in the SEM; SE = standard error; Std.lv = standardized estimate of the continuous latent variable.

^a^Latent factor of the one-factor model (positive and negative mental health as two opposite poles).

## Discussion

One aim of this study was to analyze a multidimensional assessment of strengths developed for the application in clinical samples. Many patients experience a lot of negative feelings and low self-efficacy in dealing with current problems at the beginning of psychotherapy (Tecuta et al., 2015). As studies suggest, the perception of one's own strengths also seems to be limited by this negative perspective. Strengths that are present despite the problems and symptoms (e.g., taking up a hobby) are not necessarily experienced by patients as helpful, although outsiders would name these aspects as strengths. Only measuring strengths to deal with current problems seems to provide little information gain in the clinical context, as such measures tend to inversely express problem burden. The assessment tool used in this study (i.e., the WIRF) measured strengths with three subscales: (1) strengths in everyday life (EvdayS), (2) strengths used to successfully cope with previous crises (CrisesS), and (3) strengths in connection with current problems (ProbS). It was intended to examine whether the subscales are indeed distinguishable and whether they provide a better prediction of mental health. Another aim of this study was to test the assumptions of the dual-factor model of mental health on another clinical sample. For this purpose, we investigated whether patients' data at therapy start point to an independence of well-being and distress, or whether only one of these states was experienced at a time.

Results showed that the WIRF subscales were significantly interrelated with moderate to large coefficients. ProbS showed moderate correlation coefficients in relation to PMH and NMH measures, while EvdayS and CrisesS were only slightly associated with these variables. Although each subscale was comprised of the identical 12 items, the three-subscale solution of the WIRF was confirmed. The subscales were filtered out as partially independent factors, suggesting that strengths can be captured in separate contexts by using explicit instructions. Only a one-factor model of mental health/illness was appropriate for data of the clinical sample. NMH measures were positively related, and PMH measures negatively related to the latent factor. This result means that patients with high symptom burden hardly experienced well-being at the same time. All WIRF subscales were significant predictors of the mental illness factor in the latent regression analysis. The coefficients of WIRF-CrisesS and WIRF-ProbS remained stable in the multiple regression analysis. These two subscales were significant and incremental predictors of lower mental illness.

### Interpretation of Results

Our first hypothesis was confirmed as findings support the multidimensional structure of the WIRF. Although all subscales query the same 12 items and the same strengths in terms of content, they could be statistically distinguished. The questionnaire uses instructions to focus patients' perceptions on the particular context. In contrast, established instruments only capture positive trait characteristics or strengths that are currently experienced (Peterson & Park, 2009; Tagay et al., 2014). A unique feature of the instrument in this study is that the WIRF also captures strengths that have been used successfully in the past and in good times. This differential assessment of strengths seems to be relevant in clinical samples, as studies indicate a high problem focus and negative affect in patients (Stanton & Watson, 2014). Willutzki (2008) states that the high level of suffering of individuals at the beginning of therapy leads to the fact that they hardly perceive existing strengths in themselves or evaluate them as helpful. In other words, patients’ perception of their strengths is strongly related to current distress and can hardly be assessed independently of problems (cf. Iasiello et al., 2022). The statistically independent subscales of the WIRF may make existing strengths more visible to patients themselves and their therapists. This might have scientific implications: As shown in the testing of the third hypothesis, the WIRF subscales were independent predictors of mental illness. WIRF-ProbS accounted for the largest proportion of variance, which means that a person with many self-perceived strengths for coping with current problems had fewer symptoms and more well-being. This result was to be expected since successful problem management usually leads to less stress. Beyond this effect, WIRF-CrisesS incrementally predicted mental illness. This indicates that patients who are currently under a lot of stress, but at the same time know what strengths have helped them in the past, have better mental health in comparison to persons with less good strengths awareness. The awareness of strengths in coping with previous crises may be associated to a stable sense of mastery, which was positively related to resilience and mental health in prior studies (Burns et al., 2011). WIRF-CrisesS may be relevant to research that focuses on the description and etiology of mental health in clinical populations, as it seems to be less entwined with psychopathology and, therefore, may contribute to an increase in information (Bos et al., 2016). Moreover, in the context of psychotherapy research, WIRF-CrisesS was found to be a significant predictor of treatment outcome beyond problem-associated measures (Schürmann-Vengels et al., 2022).

The independence of WIRF subscales also provide practical implications: Although recent studies indicated that patients perceive fewer current strengths than healthy individuals, this does not mean that strengths to cope with their problems do not exist (Goldbach et al., 2020; Victor et al., 2019). The results of this study highlight that it makes sense for therapists to actively address existing strengths to further foster mental health. It may be helpful to draw the patient’s attention to helpful abilities, pleasant activities, or positive relationships. For example, interventions from the solution-focused brief therapy are recommended because these target situations in which patients have already been able to use their strengths successfully (similar to WIRF-CrisesS; Franklin et al., 2017). The diagnostic of strengths during treatment with the WIRF can have the advantage that patients on the one hand recognize which strengths have helped them in the past (via CrisesS) and on the other hand experience how strengths develop during psychotherapy (via ProbS). Patients answered the subscales differently in this study, which suggests that a comparison between the contexts may provide therapists with additional information. This could facilitate working with patients’ strengths in sessions.

The dual-factor model of mental health was not supported in this clinical sample. A high association of positive and negative variables was found, similar to prior studies in this framework (Franken et al., 2018; Lukat et al., 2016; van Erp Taalman Kip & Hutschemaekers, 2018). This finding suggests that positive and negative facets of mental health are more entwined in people with pronounced symptoms than in healthy subjects. One possible explanation for this finding could be that patients focus strongly on burdensome factors at the beginning of psychotherapy. From a clinical perspective, such negativity bias may contribute to patients' poorer ability to perceive positive aspects in their lives or to judge them as relevant (Carl et al., 2013; Gollan et al., 2016). This, in turn, might lead to patients frequently talking about problems and little about positive experience in the therapy session. A recent study also showed that instruments assessing PMH are answered differently by individuals with severe distress than by healthy subjects (Iasiello et al., 2022). Patients may tend to condition their well-being on the presence of psychopathological symptoms and automatically fill out positive questionnaires low. These explanatory attempts should be considered as hypotheses and tested in future research.

Almost all studies on the dual-factor model find degree of independence of positive and negative facets of mental health even in clinical samples (de Vos et al., 2018; Díaz et al., 2018; Franken et al., 2018; Teismann et al., 2018). In addition, a study using ecological momentary assessment in individuals with generalized anxiety disorder showed that these people self-reported several positive phases in their daily lives, despite severe worry (Vîslă et al., 2021). These results suggest that patients can, in principle, also report well-being and positive moments. However, a problem focus often dominates in patients themselves and in therapy. Therefore, it is recommended to provide space for positive reports from patients (even if they are rare or seem small). Therapists should also ask specifically about patients’ strengths, exceptions, and positive changes.

### Limitations and Future Directions

This study has several limitations. The size of the clinical sample was small for SEM, according to established thumb rules of 5-10 observations per parameter, so that replication studies are needed. On the other hand, simulation studies indicated that even smaller sample sizes could be sufficient for particular SEM analyses (e.g. Wolf et al., 2013). No comparisons to other clinical samples or healthy controls were included, which limits generalizability of the results. Moreover, the cross-sectional design restricted the predictive value assumed in the regression analysis. Longitudinal designs should analyze the predictive relevance of the strengths subscales for PMH and NMH. Furthermore, moderation analyses should differentiate how resources act on mental health in clinical samples. Our results suggest the assessment of strengths in psychotherapy studies. Repeated assessment of strengths during treatment should trace potential increases of PMH and related process factors.

### Conclusion

The WIRF is a promising complementary instrument of strengths in clinical psychology and psychotherapy. Its multidimensional structure reaching beyond current problems is a unique feature of the instrument and may be relevant for etiology and intervention studies. The results of this study suggest that PMH is not easily detected in the presence of simultaneous marked psychopathology. This underlines the relevance of differential assessments of patients’ positive facets.
